# Supplementary material for: Role and mechanism of NCAPD3 in promoting malignant behaviors in gastric cancer
Source: Front Pharmacol. 2024 Apr 22;15:1341039. doi: 10.3389/fphar.2024.1341039 (PMC11070777; doi:10.3389/fphar.2024.1341039)
Supplement: Supplementary file 11 [file DataSheet2.ZIP › GSEA/immunologic/my_analysis.Gsea.1599462003817/GSE14415_NATURAL_TREG_VS_FOXP3_KO_NATURAL_TREG_DN.html]

Details for gene set GSE14415\_NATURAL\_TREG\_VS\_FOXP3\_KO\_NATURAL\_TREG\_DN[GSEA]

|  || Dataset | filtered\_dataset.sample\_info.cls#WT\_versus\_NCAPD3\_MUT |
| Phenotype | sample\_info.cls#WT\_versus\_NCAPD3\_MUT |
| Upregulated in class | WT |
| GeneSet | GSE14415\_NATURAL\_TREG\_VS\_FOXP3\_KO\_NATURAL\_TREG\_DN |
| Enrichment Score (ES) | 0.4677894 |
| Normalized Enrichment Score (NES) | 1.8827726 |
| Nominal p-value | 0.0034364262 |
| FDR q-value | 0.5615728 |
| FWER p-Value | 1.0 |
Table: GSEA Results Summary

  

Fig 1: Enrichment plot: GSE14415\_NATURAL\_TREG\_VS\_FOXP3\_KO\_NATURAL\_TREG\_DN      
 Profile of the Running ES Score & Positions of GeneSet Members on the Rank Ordered List

  

| SYMBOL | TITLE | RANK IN GENE LIST | RANK METRIC SCORE | RUNNING ES | CORE ENRICHMENT || 1 | 51111 | KMT5B | 2 | 1.217 | 0.1063 | Yes |
| 2 | 54894 | RNF43 | 29 | 1.000 | 0.1761 | Yes |
| 3 | 23253 | ANKRD12 | 34 | 0.977 | 0.2598 | Yes |
| 4 | 375341 | C3orf62 | 49 | 0.919 | 0.3311 | Yes |
| 5 | 152877 | FAM53A | 67 | 0.857 | 0.3947 | Yes |
| 6 | 153364 | MBLAC2 | 79 | 0.838 | 0.4610 | Yes |
| 7 | 2186 | BPTF | 159 | 0.717 | 0.4678 | Yes |
| 8 | 84674 | CARD6 | 463 | 0.497 | 0.2944 | No |
| 9 | 3434 | IFIT1 | 743 | 0.354 | 0.1257 | No |
| 10 | 10123 | ARL4C | 957 | -0.377 | 0.0062 | No |
| 11 | 493869 | GPX8 | 966 | -0.385 | 0.0345 | No |
| 12 | 51646 | YPEL5 | 1014 | -0.413 | 0.0374 | No |
| 13 | 857 | CAV1 | 1098 | -0.475 | 0.0199 | No |
| 14 | 5359 | PLSCR1 | 1146 | -0.511 | 0.0314 | No |
| 15 | 27250 | PDCD4 | 1170 | -0.537 | 0.0625 | No |
| 16 | 4189 | DNAJB9 | 1223 | -0.598 | 0.0781 | No |
| 17 | 114907 | FBXO32 | 1248 | -0.625 | 0.1162 | No |
Table: GSEA details [plain text format]

  

Fig 2: GSE14415\_NATURAL\_TREG\_VS\_FOXP3\_KO\_NATURAL\_TREG\_DN      
 Blue-Pink O' Gram in the Space of the Analyzed GeneSet

  

Fig 3: GSE14415\_NATURAL\_TREG\_VS\_FOXP3\_KO\_NATURAL\_TREG\_DN: Random ES distribution      
 Gene set null distribution of ES for **GSE14415\_NATURAL\_TREG\_VS\_FOXP3\_KO\_NATURAL\_TREG\_DN**
